# One Decade Later: What has Gene Expression Profiling Told us About Neuronal Cell Types, Brain Function and Disease?

**DOI:** 10.2174/138920209788921029

**Published:** 2009-08

**Authors:** Elva Díaz

**Affiliations:** UC Davis School of Medicine, Department of Pharmacology, Davis, CA 95616, USA

**Keywords:** Microarray, expression profiling, brain disease, transcriptome, neuronal cell type.

## Abstract

The many diverse functions executed by the central nervous system (CNS) are mirrored in the diverse shapes, connections, and firing patterns of its individual neuronal cell types. Furthermore, distinct neurological diseases are the result of defects in specific neuronal cell types. However, despite the significance of this cellular diversity underlying brain function and disease, we know relatively little about the genes that contribute to purposeful differences among regions and cell types within the brain. A major challenge in this endeavor is the paucity of markers that define the many regions and cell types thought to exist. Cataloging the neuronal cell types and cell- and region-specific marker genes requires novel avenues that enable researchers to define gene expression profiles of brain regions and individual neurons and to apply this information to understand functional and structural properties in the normal and diseased brain. Functional genomic approaches such as gene expression profiling offers the exclusive opportunity to glimpse the detailed inner workings of distinct neuronal cell types. Recent studies have applied microarray technology in unique and novel ways to understand the molecular mechanisms that underlie such neuronal diversity and their potential role in brain diseases.

## INTRODUCTION

A major challenge in understanding brain development and function is the enormous complexity of the central nervous system (CNS). The complexity of the mammalian brain is reflected in the diverse cell types of its neuronal constituents - their vastly different morphologies, synaptic connections and functional properties. Recent reviews have discussed in detail the working definition of a neuronal cell type based on various criteria [1, 2] and will not be reiterated here. That different neurological diseases result from distinct perturbations of specific cell types in the CNS further illustrates the precise and highly specialized nature of its neuronal components. For example, in Parkinson’s disease midbrain dopaminergic neurons of the substantia nigra are acutely sensitive to degeneration in the adult while in the pediatric brain tumor medulloblastoma granule neuron precursors become transformed during development of the cerebellum. In each brain disorder, specific genetic mutations are thought to be etiologic in the development of the disease. Clearly, no two neuronal populations are created equal. How is one to ever make sense of this daunting complexity in order to understand and work towards cures for diverse brain diseases?

Since the introduction of microarray technology and the establishment of the field of functional genomics almost fifteen years ago [3-5], researchers around the world have applied this powerful technology to define the ‘transcriptome’ underlying diverse biological phenomena from metabolic growth of single-cell organisms [6] to complex interactions between host and virus during infection [7] whereby one can monitor gene expression on a genome-wide scale in a single experiment. At first glance, microarray technology seemed uniquely poised to tackle fundamental questions in the neurobiology of disease [8]. However, it has taken a longer time for this technology to pervade the neuroscience community due to the complex nature of brain tissue and the need to overcome technical obstacles such as amplification of starting material from small target areas, a fact that was recognized early on [9,10]. While the first study to amplify linearly transcripts from single neurons was published almost two decades ago [11], it took some time to optimize this method for microarray-based genome-wide expression studies that require high yields of amplified material. In spite of these potentially insurmountable obstacles at that time, the power of the microarray as a mechanism to unravel the complexities of the brain was immediately recognized. Indeed, an early editorial in Nature Neuroscience suggested that two of the most important applications for microarray technology in the brain were to identify differences between brain regions and cell types that determine their specific functions and connectivity patterns and to document abnormal patterns of gene expression in animal models of brain disorders that are etiologic in disease [12]. How close (or yet how far) have we come to realizing these goals? Interestingly, compared to one decade ago when there were only two published studies by neurobiologists using microarrays there are now almost two thousand references in PubMed with the search criteria ‘microarray’ and ‘brain’. Clearly, microarray technology is much more accessible to the neurobiology community but have we been able to answer fundamental questions regarding the role of genes and their expression patterns in brain function and disease to a significant extent? To put it bluntly, what has gene expression profiling in the brain done for us lately?

In answering this question, it is critical to understand why gene expression profiling in the brain is so important in the first place. The human genome contains approximately ~30,000 genes [13,14] the function of which for the vast majority is unknown. Early evidence in rodent models indicated that the brain has increased complexity of gene expression compared with any other region of the organism (see [15] for review). These early findings have been supported by analysis of large-scale sequencing projects of human brain cDNA libraries in which approximately one third of all genes are expressed preferentially in the brain. Because clustering of gene expression profiles groups together efficiently genes of known similar function [16], not only will identifying genes and gene expression patterns that underlie this complexity of neuronal cell types provide invaluable insight in the fundamental organization of the mammalian CNS but coregulated expression of genes of known function with poorly characterized or novel genes provides a first step in the assignment of specific functions of the many genes expressed in the brain for which information is yet not available. Together, these methods will allow us to begin to unravel the mysteries of how the dysfunction of specific genes and neuronal cell types might lead to diseased states of the brain.

In the past few years, significant progress has been made in our understanding of brain organization by the application of functional genomic approaches to characterize gene expression in defined cell types [1]. In general, such approaches rely on the labeling and isolation of defined neuronal cell types, the amplification of genetic material, and identification of differentially expressed genes (reviewed in [2]). In addition, other larger scale approaches not discussed in detail here have been complementary to our forward progress in understanding of gene expression underlying neuronal complexity including the large-scale transgenic-based gene expression atlas of the CNS based on bacterial artificial chromosomes (GENSAT [17]) and the genome-wide Allen Brain Atlas of gene expression in the adult mouse brain [18]. These expression based approaches coupled with large scale functional approaches such as the trans-NIH initiative Knockout Mouse Project (http://www.knockoutmouse.org/) to knock-out every gene in the mouse genome will provide an amazing database of information to mined with regard to understanding candidate genes from expression profiling approaches in brain function and disease.

Below I provide a review of published studies analyzing gene expression in defined neuronal populations or specific brain regions with emphasis on recent studies that have led to the identification and characterization of candidate genes involved in brain function and disease – a goal for which expression profiling was expected to have a major impact a decade ago. In addition, I highlight novel approaches to analyze gene expression data and follow-up studies that have moved beyond the usual end-point of generating lists of candidate genes.

## EXPRESSION PROFILING OF BRAIN REGIONS IN NORMAL AND DISEASED TISSUES

From the beginning, reports of gene expression profiling in human brain tissues revealed interesting insights into diseases of the brain. For example, gene expression profiling of prefrontal cortical tissue from age-matched pairs of schizophrenic and control subjects showed a decrease in messages encoding proteins involved in the regulation of presynaptic function correlated with the schizophrenia subjects [19]. The basic assumption for this and other studies is that variation in gene expression is an important mechanism underlying susceptibility to complex diseases such as schizophrenia. This study led to the demonstration that the transcript encoding regulator of G-protein signaling 4 (RGS4) was consistently and significantly decreased in the prefrontal cortex of all schizophrenic subjects examined [20], suggesting that decreased RGS4 expression thereby affecting neuronal signaling might be a distinct feature of schizophrenia. Satisfyingly, initial evidence for linkage with schizophrenia was reported near RGS4 and several association studies also suggested modest associations for certain RGS4 gene variants (see [21] for one association study example). However, follow-up studies have yielded conflicting and even negative results, which has complicated the significance of this candidate gene. A possible explanation of the discrepancies is that RGS4 variants might modulate endophenotypes (measurable components expressed as quantitative traits along the pathway to development of the full-blown disease) associated with schizophrenia rather than risk of disease itself [22]. For instance, certain RGS4 polymorphisms appear to contribute to structural alterations in brain areas previously associated with schizophrenia [23, 24]. While we are far from a clear understanding the potential role of RGS4 in schizophrenia, its identification as a candidate gene with gene expression profiling demonstrates the potential impact of applying microarray technology to the brain but highlights the need for extensive follow-up studies to characterize the function of the candidate genes identified in the biological processes in which they are implicated.

In general, gene expression studies aimed at identifying transcripts present in subsets of neurons or expressed at low levels are complicated by the cellular heterogeneity of the brain [25]. For example, an early study by Sandberg and colleagues analyzed baseline gene expression differences in six brain regions (cortex, hippocampus, amygdala, entorhinal cortex, midbrain, and cerebellum) and found that the histologically less complex cerebellum showed many more uniquely expressed genes than the cerebral cortex or hippocampus [26] most likely due to the detection limit of microarray experiments at that time. Moreover, a larger problem exists in that the expression profile of any given gene from a complex tissue such as the brain reflects the transcript level within most likely more than one cell type.

This general issue led to the application of biostatistical approaches such as linear modeling and regression analysis of microarray data to ‘deconvolute’ expression profiles derived from whole brain regions [27]. In this study, the authors analyzed gene expression in the developing mouse pontocerebellar projection system in wild type animals and in mouse lines with mutations known to affect specific cell types in the cerebellum. The expression profile for each gene was calculated by linear combination of the regression output resulting in the ‘X statistic,’ that is, a quantitative measure of gene expression differences in specific cell types [27]. The combination of gene expression profiling and genetic mutant analysis coupled with sophisticated statistical analysis allowed the ability to dissect gene expression programs underlying differentiation of specific cell types (Purkinje neurons, granule neurons, and glia) within a complex brain tissue, the cerebellum.

Ultimately, this study identified groups of genes representing the early specification of granule neuron precursors (GNPs), the proliferation of this cellular pool, and finally the later stages of granule neuron differentiation [27]. While several genes were already known to function in granule neuron development providing important validation of the approach, a large number of genes had not been previously implicated in neural development. For example, expansion of the GNP pool occurs during early postnatal development and is regulated by Nmyc [28, 29] as part of the sonic hedgehog (Shh) signaling pathway [30]. The approach by Diaz and colleagues identified genes transiently upregulated during GNP proliferation with profiles similar to those of known Shh target genes such as Cyclin D2 [27]. One of these genes with a high X-statistic value encoded the transcription factor Mad3, a member of the Myc/Max/Mad family of transcriptional regulators [31]. Recent evidence born out of the expression profiling approach demonstrated a novel role for Mad3 in promoting Shh-mediated GNP proliferation [32], providing important functional validation of the microarray study. This study is particularly noteworthy in light of current models for Mad function that postulate that Mad proteins promote *differentiation* by antagonizing Nmyc-mediated proliferation [33]. That is, the unbiased nature of the expression profiling approach predicted a role for Mad3 in a biological pathway that would not have been expected otherwise.

With regard to brain disease, previous studies showed that aberrant Shh signaling contributes to cerebellar tumors in both mice and humans [34] and Nmyc is an essential downstream effector of Shh signaling during both normal and neoplastic cerebellar growth [35]. Interestingly, Mad3 is upregulated in mouse models of medulloblastoma [32] as well as human medulloblastoma samples [36], suggesting that like Nmyc (which coincidently was initially identified in an expression profiling study of purified GNPs treated with Shh [29]), Mad3 might play a role in tumor biogenesis. Further studies with genetically modified animals will be necessary to test this possibility directly.

More recently, other bioinformatics methods such as systems biological approaches have been developed to identify groups of genes, or ‘modules’, with highly correlated expression levels in brain regions [37]. To define these modules, the authors used a statistical approach called weighted gene coexpression network analysis (WGCNA) in which a pair-wise correlation matrix is calculated for all expressed genes and then these correlation measurements are weighted to determine the coexpression strength between genes with the final output being a transcript network where genes are grouped based on their dependence on each other [38]. In contrast to the identification of differentially expressed genes *between* brain regions, this strategy takes advantage of the inherent variability associated with gene expression profiles that exist *within* brain regions – that is, biological replicates from a single brain region - to define higher-order relationships among gene products. Using this approach, Oldham and colleagues defined gene coexpression relationships in microarray studies generated from specific human brain regions. Comparison of conserved gene modules from different brain regions then led to the identification of coexpressed genes that correspond to distinct cell types including neurons, oligodendrocytes, astrocytes and microglia [37], demonstrating that cell type-specific information can be identified from whole brain tissue with this approach.

The application of WGCNA to normal and diseased brain might then provide a means to reveal biological relationships of the phenotypic source of the observed transcriptome changes not apparent with traditional analysis of differential gene expression. For example, despite the fact that Alzheimer’s disease is the most common and well studied neurological disorder of the elderly, no coherent picture of the pathology underlying this disease has emerged from previous microarray expression studies in part due to artifacts associated with collection of postmortem samples or with different microarray platforms often resulting in non-overlapping gene lists [8]. Miller and colleagues applied WGCNA to existing expression data composed of postmorten tissue during Alzheimer’s disease progression and identified twelve distinct coexpression modules implicated in synaptic function, immune response, and metabolism [39]. Comparison with gene expression data from normal aging in the brain allowed the identification of modules conserved between the two states [39], suggesting that common pathophysiological processes underlie aspects of Alzheimer’s disease progression and normal aging. A particular strength of the approach is the identification of ‘hub’ genes that occupy key positions in coexpression modules correlated with Alzheimer’s disease progression [39]; thus, understanding the role of such hub genes in follow-up studies might provide novel insights into the disease as well as their role in normal aging. However, while the WGCNA modules identified are currently limited to the major cell classes in the brain, expansion of the WGCNA analysis to include large more precise transcriptome profiles corresponding to isolated populations of neurons from normal and diseased samples should allow the refinement of the existing modules into neuronal subtype-specific classifications in normal brain and during disease progression.

## EXPRESSION PROFILING OF ISOLATED NEURONAL CELL TYPES IN NORMAL AND DISEASED BRAIN

In addition to bioinformatics approaches, other avenues of research were applied early on to understand gene expression in neuronal cell types. Early reports were successful in generating expression profiles from single neurons isolated by laser capture microdissection (LCM) for microarray expression analysis [40, 41]; however, the overall quality of the expression profiling dataset decreases substantially. Recent advances have seen the development of new techniques for reliable and reproducible analysis of single cells. Esumi and colleagues developed a combined PCR and T7 RNA polymerase amplification technique for gene expression analysis at the single cell level [42]. With this approach, the authors analyzed single GABAergic neurons progenitors isolated from the neocortex of GAD67-GFP knock-in mice by dissociation and aspiration of GFP-positive cells and demonstrated robust results from microarray expression analysis.

An alternative strategy to circumvent the need to analyze individual cells is to isolate pools of defined neuronal subtypes labeled by transgenic or tracer injection methods (reviewed in [2]). For instance, Sugino and colleagues carried out microarray expression analysis of twelve populations of defined neuronal cell types labeled with fluorescent proteins in transgenic mice or with stereotaxic injection of fluorescent tracers and then isolated cells from adult mouse forebrain by manual sorting techniques [43]. Using these expression profiles, the authors were able to generate a dendrogram that reflected the expected major subdivisions between these populations, such as the distinction between cortical interneurons and projection neurons. The strength of this approach is that it relies on a single measurement, gene expression distance, to incorporate information over a wide range of cellular functions and can be used to compare any two neuronal populations on a defined set of variables. In addition to the classification of unknown neuronal subtypes, this dataset should also be useful for the identification of neuronal subtypes specifically altered during disease progression. Indeed, by measuring the strength of the gene expression distance between normal neuronal cell types and cells during disease progression one might be able to identify specific biomarkers corresponding to distinct phases of disease progression that could serve a clinical value.

In addition, other methods such as fluorescent activated cell sorting (FACS) have been used successfully to isolate labeled neuronal cell types (reviewed in [2]). For example, an elegant study by Arlotta and colleagues used FACS to isolate corticospinal motor neurons (CSMN) retrogradely labeled by injecting microspheres coated with fluorescent tracer into their axonal projection fields at distinct stages of development *in vivo* and compared their gene expression to two other pure populations of cortical projection neurons: callosal projection neurons and corticotectal projection neurons [44]. The identified gene expression profiles predicted which genes might play instructive roles in CSMN development. Indeed, the authors demonstrated that one of the newly identified candidate genes (Ctip2) plays a critical role in the development of CSMN axonal projections to the spinal cord with loss-of-function experiments in null mutant mice for Ctip2 [44], providing impressive validation of their experimental approach to identify key genetic determinants of the CSMN population.

In more recent studies, these same authors then went on to show that Ctip2 also plays a role in striatal medium spiny neurons (MSN) differentiation and development [45]. MSNs are important for motor control and their degeneration is a principal aspect of Hungtington’s disease. In the striatum of Ctip2 null mice, MSNs exhibit defects in neuronal differentiation as evidenced by decreased expression of known MSN marker genes [45]. Interestingly, the cellular architecture of the striatum is dramatically altered as MSNs fail to form into a patch-matrix organization, thereby leading to abnormal innervation of the striatum by dopaminergic inputs [45]. While the role of Ctip2 in Huntington’s disease itself is unknown, its role in normal development of MSNs warrants further study of this gene in progression of this disease.

Other studies have pursued similar methods to isolate defined neuronal populations using FACS but with different methods to label the cells (reviewed in [2]). For example, Lobo and colleagues developed a method to purify genetically labeled neurons from the GENSAT BAC transgenic mice for gene expression profiling [46]. Using this approach, the authors identified a new set of differentially expressed genes in the striatonigral and striatopallidal neurons from juvenile and adult mice, two functionally and clinically important projection neuron subtypes in the basal ganglia. Importantly, the authors provided functional validation of their expression profiling approach by demonstrating that Ebf1 is a lineage-specific transcription factor essential to the differentiation of striatonigral neurons [46].

The results of this study might impact brain disorders of the basal ganglia by the identification of candidate genes to follow-up on in future studies. Interestingly, two of the most promising identified genes (Dock3 and Slc35d3) have some known association with movement disorders. Dock3 has been implicated in one family with an attention deficit and hyperactivity disorder (ADHD)-like clinical syndrome [47] while Slc35d3 might function in striatonigral-specific protein glycosylation potentially implicated in the childhood hyperkinetic movement disorder Sydenham chorea of which the pathophysiology is linked to antibodies that recognize sugar moieties of glycoproteins on MSNs [48]. While this study only provides an initial suggestion of a potential impact into our understanding of movement disorders, the identification of these and other candidate genes clearly deserves further study.

A common concern for using approaches such as FACS and LCM is that these cell isolation techniques themselves might actually introduce variability into the resulting expression data. It will be useful therefore to compare the results from studies utilizing different approaches to isolate samples but from the same brain region and disease. For example, Parkinson's disease is caused by a progressive loss of the midbrain dopamine (DA) neurons in the substantia nigra. Moran and colleagues analyzed brain tissue from clinically well-documented and pathologically documented cases of sporadic Parkinson's disease to define expression profiles corresponding to medial and lateral substantia nigra [49]. After extensive brain tissue-based validation and additional data analysis, the authors then refined their analysis to identify a list of 892 highly dysregulated ‘priority genes’ hypothesized to form the core of the diseased Parkinsonian metabolic network [50].

In contrast, Simunovic and colleagues used LCM to isolate dopaminergic neurons from the substantia nigra of control subjects and individuals with idiopathic Parkinson's disease matched for age and postmortem interval followed by microarray analysis to document gene expression changes [51].

Both studies identified genes previously implicated in Parkinson’s disease based on published literature [50, 51]. In terms of new biological insights into the progression of Parkinson’s disease, the study by Moran and colleagues identified biological associations of Parkinson’s disease with cancer, diabetes and inflammation [50] while the study by Suminovic and colleagues implicated genes involved in synaptic activity such as ion channel receptors [51]. However, the only overlap between these two studies was the dysregulation of multiple genes associated with programmed cell death [50, 51]. Because neither of these studies demonstrated a functional role for an identified candidate gene implicated in Parkinson’s disease, it is premature to declare which approach is more robust. Indeed, since the application of WGCNA to large datasets appears to be independent of platform and sample collection, this approach might provide a way to unify the various candidate gene lists generated by expression profiling in these and other studies conducted during the past decade.

## OTHER APPROACHES FOR ANALYZING GENE EXPRESSION IN NEURONAL CELL TYPES

A particularly interesting idea is to molecularly ‘tag’ the mRNA population within a defined neuronal subtype to allow selective analysis of a particular cell type of interest. For example, Von Stetina and colleagues profiled gene expression throughout the nervous system in worms by generating a stable, chromosomally integrated transgenic line expressing an epitope-tagged poly-A binding protein throughout the nervous system [52]. Such tagged mRNA could then be isolated by immunoprecipitation for cell-specific transcripts. Unfortunately, this approach would not be feasible for studies utilizing postmorten human brain tissue. However, this approach could readily be adapted to mice by taking advantage of large-scale transgenic approaches such as GENSAT [17] and the Allen Brain Atlas [18] to express epitope-tagged poly-A binding protein under cell type specific promoters and then crossing these transgenic animals with mouse models of brain disease.

In addition, deep sequencing methods allow direct ultra-high-throughput sequencing of RNA, which can then be mapped back to the genome (reviewed in [53]). However, it is important to keep in mind that these sequencing machines produce terabytes of data on a daily basis, and thus, make profound demands on bioinformatic capabilities for data storage and assembly of sequence information for individual labs. Furthermore, the inherent short reads generated by these methods pose significant problems for the interpretation of transcripts arising from gene families with high homology or repetitive regions of the genome, a problem that will be particularly evident in the brain with numerous family members expressed in distinct and overlapping subsets of neuronal cell types. Nevertheless, it can be anticipated that by the end of this decade, if not sooner, many studies will rely on this technology for large-scale analysis of gene expression in the brain.

## CONCLUSIONS AND FUTURE DIRECTIONS

The past decade has witnessed three orders of magnitude increase in the information available regarding microarrays and gene expression in the brain. While we have just begun to make significant inroads into the characterization of candidate genes identified in the various expression profiling studies, clear evidence emerges demonstrating the potential impact of these and other approaches. However, moving from gene lists, coexpression modules, or ‘hub’ genes to function in brain disorders still remains a major challenge in most expression profiling studies. Transcriptome studies by definition are correlation based, not causative. Thus, researchers pursuing expression studies should first have a plan mapped out for future research to determine how one will demonstrate function of potential candidate genes identified in their experiments.

A particularly important avenue of future research is the simultaneous genome-wide analysis of gene expression and genetic variation to map genetic factors that underlie individual differences in quantitative levels of expression (expression QTLs or eQTLs; [54]). In the eQTL method, gene expression profiles are treated as quantitative traits and genome-wide association or linkage mapping is performed to localize regulatory elements that affect the expression of the corresponding differentially expressed gene. The underlying rationale is that if a regulatory element coincides with the known location of the differentially expressed gene, it most likely represents a cis-acting regulatory element, whereas a regulatory element identified at a different location most likely represents a trans-acting regulatory element. The availability of systematically generated eQTL information could provide immediate insight into a biological basis for disease associations identified through association studies, and might allow the identification of gene networks underlying disease pathogenesis.

Even though to date there are no publications utilizing this methodology to identify genes that influence brain disease progression, a modification of this method has been applied to mouse inbred strains to identify genes that influence the volume of the amygdala, a brain region that regulates emotion [55]. In this work, amygdala volume was first quantified across various mouse strains and traditional QTL mapping was then carried out by linkage analysis to identify loci that affect phenotype. Next, to prioritize the search for candidate genes located within a linkage interval, whole brain gene expression levels of the studied strains were treated as complex traits, and their covariance with the neuroanatomical traits was analyzed to identify genetic loci that influence the expression of differentially expressed genes [55]. Several genes were found to have expression levels that correlate with the size of the amygdala across the studied strains [55], suggesting that they can be considered as possible candidate genes that regulate the anatomical phenotype. Therefore, by assessing which genes have expression patterns that correlate with brain disease and by mapping regulatory elements for these differentially expressed genes, it might be possible to find disease related regulatory networks in specific brain regions. However, since many regulatory networks are highly brain region specific it will be important to conduct eQTL mapping studies using data from brain regions or isolated cell types that are physiologically and phenotypically relevant to the trait of interest as those methods described in this review.
